# Topical hydrogel containing *Fumaria vaillantii* Loisel. extract enhances wound healing in rats

**DOI:** 10.1186/s12906-019-2645-y

**Published:** 2019-09-12

**Authors:** Fatemeh Davoodi-Roodbordeii, Minoo Afshar, Fatemeh Haji Abas Tabrizi, Samira Choopani, Giti Torkaman, Fariborz Moayer, Mona Salimi

**Affiliations:** 1 Department of pharmaceutics, Pharmaceutical Sciences Branch, Islamic Azad University (IAUPS), Tehran, 193956466 Iran; 2 Department of Physiology and Pharmacology, Pasteur Institute of Iran, Tehran, P.O. Box 13164, Iran; 3 Physical Therapy Department, Faculty of Medical Sciences, Tarbiat Modares University, Tehran, Iran; 4 Department of Pathobiology, College of Veterinary Medicine, Karaj Branch, Islamic Azad University, Alborz, Iran

**Keywords:** Wound healing, *Fumaria vaillantii*, Topical hydrogel, Excision, Incision

## Abstract

**Background:**

*Fumaria* species (Fumariacea) has traditionally been used in wound healing in Iranian folk medicine. However, with the discovery of newer agents, its use has faded off into total obscurity. This study explored the wound healing potential of a gel containing 10% *Fumaria vaillantii* Loisel through topical application of total extract in a model of excisional as well as incisional wound healing in albino Wistar rats.

**Methods:**

Rats were anesthetized, and excisional skin wound was established using a sterilized surgical scissors. The animals were then treated with 10% *F.vaillantii* topical gel formulation along with the gel base. The treatments were administered once a day after the injury for 21 days. For topical treatment, the hydrogel was formulated and evaluated for chemical and physical characteristics. Histopathological analysis with hematoxylin and eosin (H&E) was used for microscopic examination of the skin tissues on 21-day-old sections of excision wound. To verify collagen formation, hydroxyproline determination was performed 21 days post wound healing. Breaking strength was determined in a 10-day-old incision wound by the uniaxial tensile test.

**Results:**

Topical administration of *F.vaillantii* gel formulation significantly enhanced skin wound closure on the 6th post-wounding day compared to both gel base and the negative control, indicating an accelerated wound healing process, while a significant difference was observed on 10th and 14th post –wound days in *F.vaillantii* treatment compared to the negative control groups. Gel formulation prepared with a 10% *F. vaillantii* extract exhibited a response in terms of wound epithelialization, angiogenesis and number of hair follicles at wound area better than the gel base on the 21st post-wound day. Application of gel base produced further advantages by increasing hydroxyproline content and collagen fiber thickness. Our results on incision wound model were supported by histopathological data indicating the role of gel base in the enhancement of breaking strength.

**Conclusion:**

Traditional use of *Fumaria* species in the skin diseases was justified in this study by revealing the increase in wound healing activity after hydrogel containing *F. vaillantii* total extract administration.

**Graphical abstract:**

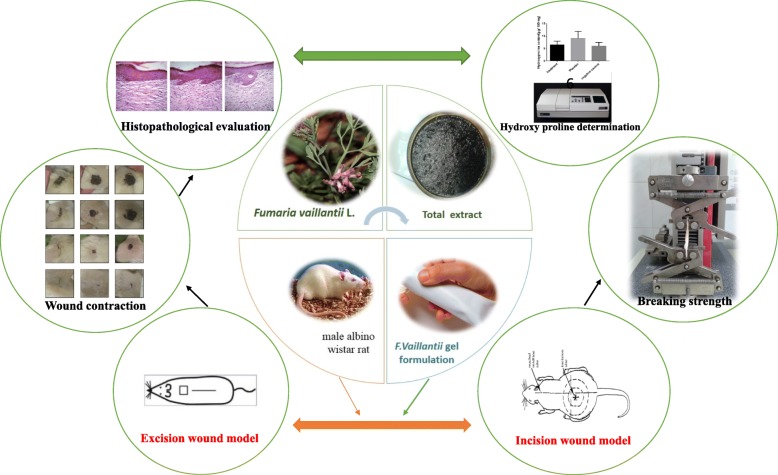

**Electronic supplementary material:**

The online version of this article (10.1186/s12906-019-2645-y) contains supplementary material, which is available to authorized users.

## Background

Wounds, which happen due to physical and chemical injuries or microbial infections, are inevitable events in life. The aim of wound healing is to bring back the structure and function of the injured tissues to the nearly pre-wound conditions. In other words, healing is an intricate body’s natural process to regenerate the integrity of damaged tissue [1–3], thus the collaborative efforts of many different tissues and cell lineages are vital for healing. Several stages are involved in a wound healing process including inflammatory, proliferation and finally remodeling phases [2, 4]. Research on drugs capable of managing each step would be of great interest in modern biomedical sciences [1]. Interestingly, up to 80% of the world’s population use herbal medicines in the treatment of various skin disorders, and known drugs obtained from plant sources have proved to enhance the healing of different wounds [1, 5]. In Iranian folklore, medicinal plants are used to heal skin wounds [6]; however, the potential of many traditional herbal extracts in wound healing still remains unexplored.

*Fumaria* genus belongs to Fumariaceae family and comprises 60 species of herbaceous flowering plants with a worldwide distribution, particularly in Asia [7]. *Fumaria vaillantii* Loisel. is one the seven species grown in different parts of Iran with the local name of “Shatareh” [8]. Aerial parts of *F.vaillantii* as an infusion have widely used by the traditional healers in the treatment of psoriasis, jaundice and fever [8]. Interestingly, the ancient Iranians used *Fumaria* spp. as a topical formulation, traditionally named “Zemad “or “Marham” to heal skin disorders [9, 10]. Most notably, native people of Jandagh, located in the central part of Iran, empirically used *Fumaria vaillantii* L. for the healing of skin wounds [6]. A vast majority of pharmacological properties including antioxidant, anti-inflammatory, anti-fungal and anti-bacterial have also been reported for this plant, which was attributed to the presence of diverse secondary metabolites [11–15]. Among a large number of compounds present in this species, alkaloids and flavonoids are two important classes [16–18]. With inflammation as an important stage involved in wound healing, *Fumaria vaillantii* possessing anti-inflammatory as well as antioxidant properties, due to the presence of metabolites, seems to be effective in the primary stages of wound repair [11, 19]. To the best of our knowledge, only a single study has been performed on the wound healing potential of *F. indica* extract [19] . To further our knowledge, the present study was undertaken to assay the wound healing property of *F.vaillantii* gel formulation in both an excision and an incision wound models in rats.

Recently, wound dressing has taken a large attraction due to creating a moist environment to support faster wound healing [20]. Amongst the existing dressings, hydrogels are reported to be optimal for use in all steps of wound healing as a drug delivery system [21]. In the present work, we aimed to formulate a hydrogel for topical administration of *F.vaillantii* extract, which is suitable for dermatological application including wound healing. To the best of our knowledge, this is a first report on the therapeutic potential of hydrogel formulation composed of *F.vaillantti* extract as a wound healing promoter in an in vivo model.

## Methods

### Plant material and extraction procedure

*F.vaillantii* plant was collected from North of Iran in August 2014. A voucher specimen (No. 6563 TEH) was deposited at the Herbarium of Faculty of Pharmacy at Tehran University of Medical Sciences and authenticated by Dr. Gholamreza Amin. Following separating the aerial parts of the plant, they were dried in the dark for 3 days. Total extract was prepared by thoroughly mixing 320 g of dried powder with ethanol: water (80:20) three times at room temperature for 72 h via maceration procedure. The extracts were evaporated to dryness and then kept at 4 °C.

### Gel formulation

Weighed quantities of different thickening agents including HPMC (Hydroxypropyl methylcellulose) 4000 cP_,_ HPMC 15 cP and Carbomer 940, which were kind donations of Hakim Pharmaceutical Company, were separately added to distilled water and allowed to soak for 24 h. The hydroalcoholic extract of *Fumaria vaillantii* (10%) was solubilized in propylene glycol (Sepidaj, Iran). The latter solution was transferred to the aqueous dispersion of each thickening agents, individually at different concentrations. The mixtures were then stirred gradually to find the best formulation. Triethanolamine was added to neutralize carbomer solution.

### Evaluation of the gel

#### pH measurement

In order to determine the pH of the gel, the glass electrode was completely dipped into a solution containing 10% of the hydrogel in deionized water.

#### Rheology of the gel

Viscosity was determined at 25 °C using a Brookfield digital viscometer-RVDV-III (Brookfield, Massachusetts, USA) and spindle no. 52 at different rpm.

#### Centrifugation test

Hydrogels were centrifuged at 6000 rpm for 30 min and then examined for phase separation.

### Animals

In our study, male albino Wistar rats weighing 220–250 g were purchased from the National Animal Center (Pasteur Institute of Karaj) and maintained in a 12/12-h light–dark cycle, with food and water supplied ad libitum. Animals were treated in accordance with the guidelines approved by the animal ethics committee of Pasteur Institute of Iran (IR.PII.REC.1397.027, 2019-01-09). Animals were divided into three groups (*n* = 5). Group I: Received administration of 10% *F.vaillantii* hydrogel formulation. Group II: Received topical application of gel base. Group III: Served as the negative control. The animals were euthanized with intra-peritoneal injection of sodium pentobarbital at 60 mg/kg on the 10th and 21st days after wounding.

### Wound healing activity assessment

Excision and incision models were used to investigate the wound healing activity of gel formulation of *F.vaillantii* total extract.

### Excision wound model

Rats were anesthetized using ketamine + xylazine and the hair on the back was clipped with electric clippers. Cutaneous square wounds of 225 mm^2^ width with 2 mm depth were inflicted on the depilated ethanol-sterilized dorsal thoracic region of rats with the help of sterilized surgical scissors under a semi-aseptic condition. The *F.vaillantii* hydrogel formulation along with the gel base was topically applied once a day for 21 days. Wound photos were taken and the wound area was measured in mm^2^ by putting a transparent sheet over it. Upon tracing the wound margin by a permanent marker, the wound area was recorded using graph paper and Image J software on 2nd, 4th, 6th, 8th, 10th, 12th, 14th 16th,18th and 21st post wound days to monitor the percentage of wound closure. The percentage of wound contraction was calculated using the formula [22]: (Initial wound size – specific day wound size)/ Initial wound size × 100.

The healed wound along with the surrounding skin obtained on day 21 were excised and then dissected into two equal parts to be further examined by histopathological analysis and hydroxyproline level determination method.

### Histopathological evaluation

The healing tissues obtained from all the three groups of animals in our excision wound model were processed for histopathological analysis. Following fixation of the tissue samples in 10% formalin, samples were dehydrated using graded alcohol series. Afterwards, skin samples were cleared in xylene and then embedded in paraffin wax. Serial sections of 5 μm were prepared and stained with hematoxylin and eosin (H&E) for routine histopathological evaluation. All slides were investigated in a blinded manner by a pathologist.

### Hydroxyproline determination

Hydroxyproline contents were spectrophotometrically measured by Woessner’s method [23]. In brief, after the weighed tissue samples were hydrolyzed in 6 N HCl for 18 h at 115 °C, the residue was evaporated to dryness and remixed with a known volume of water. Following incubation of one ml of the sample with 0.5 ml of 0.05 M chloramine T solution (Sigma-Aldrich, USA) for 20 min at room temperature, 1 ml of Erlich’s solution was added and further incubated for 15 min at 60 °C. Absorbance was measured at 550 nm using a spectrophotometer (Cecil Company, UK). Hydroxyproline level was calculated from a linear standard curve and presented as μg/100 mg of dry content.

### Incision wound model

Rats were anesthetized with ketamine + xylazine and the dorsal fur of the animals was shaved whit an electric clipper to make the incision wound. Incision of 3 cm was made at least 2 cm lateral to the vertebral column and parallel to it by a sharp scalpel with sufficient care. Afterwards, the incision was closed with surgical sutures at intervals of 1 cm. The hydrogel containing 10% of *Fumaria vaillantii* L*.* total extract and the gel base were topically applied once a day, starting from the initial day for 10 days. Sutures were removed on the 8th day and breaking strength of the healed wound and normal skin were measured using uniaxial tensile test (Model Z 2.5, Zwick GmbH & Co, Ulm-Einsingen, Germany) [24] on the 10th day.

### Confirmation of quercetin in total extract by HPLC

Quercetin content was evaluated using Waters LC 600 model chromatograph (Waters, Massachussets, USA) coupled with UV detector. Separation was performed by isocratic elution at a flow rate of 1 ml/min on Acclaim™ 120 C18 analytical column (150 mm × 4.6 mm; 5 μm). Mobile phase was a mixture of 2% acetic acid in water and acetonitrile in a 2:1 ratio and the sample injection volume was 20 μl. Stock solution of total extract of *F.vaillantii* was prepared (10 mg/ 10 ml) in ethanol and then passed through a 0.45 μm membrane filter. A 370 nm wavelength was applied for analysis. Peak areas were integrated automatically by Waters Software.

### Statistical analysis

The data are expressed as mean ± SEM of at least triplicate determinations, and comparisons were based on ANOVA followed by Tukey’s post test using a GraphPad Prism 6.0 Software. A value of *p* < 0.05 was considered as statistically significant.

## Results

### Formulation and physical evaluation of the hydrogels

Among different gelling agents used, only HPMC 4000 cP (2.5%) formed a viscous mixture and the others could not increase viscosity of the formulation. Visual examination showed that the prepared *F.vaillantii* hydrogel was stable after being subjected to centrifugation.

**Fig. 1:**
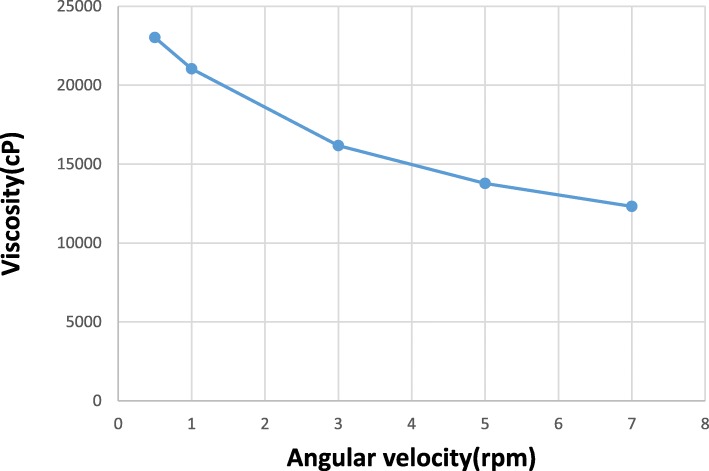
Rheological behavior of hydrogel formulation of *F.vaillantii* total extract using HPMC 4000 cP (2.5%)

Rheology is an important parameter as it affects the spreadability of the topical formulations on the skin surface. Rheological behavior of the hydrogel formulation containing HPMC 4000 cP with neutral pH of 6.4 exhibited a desirable non-Newtonian shear thinning pseudo plastic type of flow, i.e. viscosity decreases at increasing angular velocity Fig. 1.

The stability studies indicated no color fading for *F.vaillantii* prepared hydrogel 6 months after development. The pH of the hydrogel formulation was found to be within the range of 5.8–6.4, which lies in the normal pH range of the skin [25]. The rheology of the hydrogel was found to be the same after 6 months of storage at room temperature.

### Wound healing activity

**Fig. 2:**
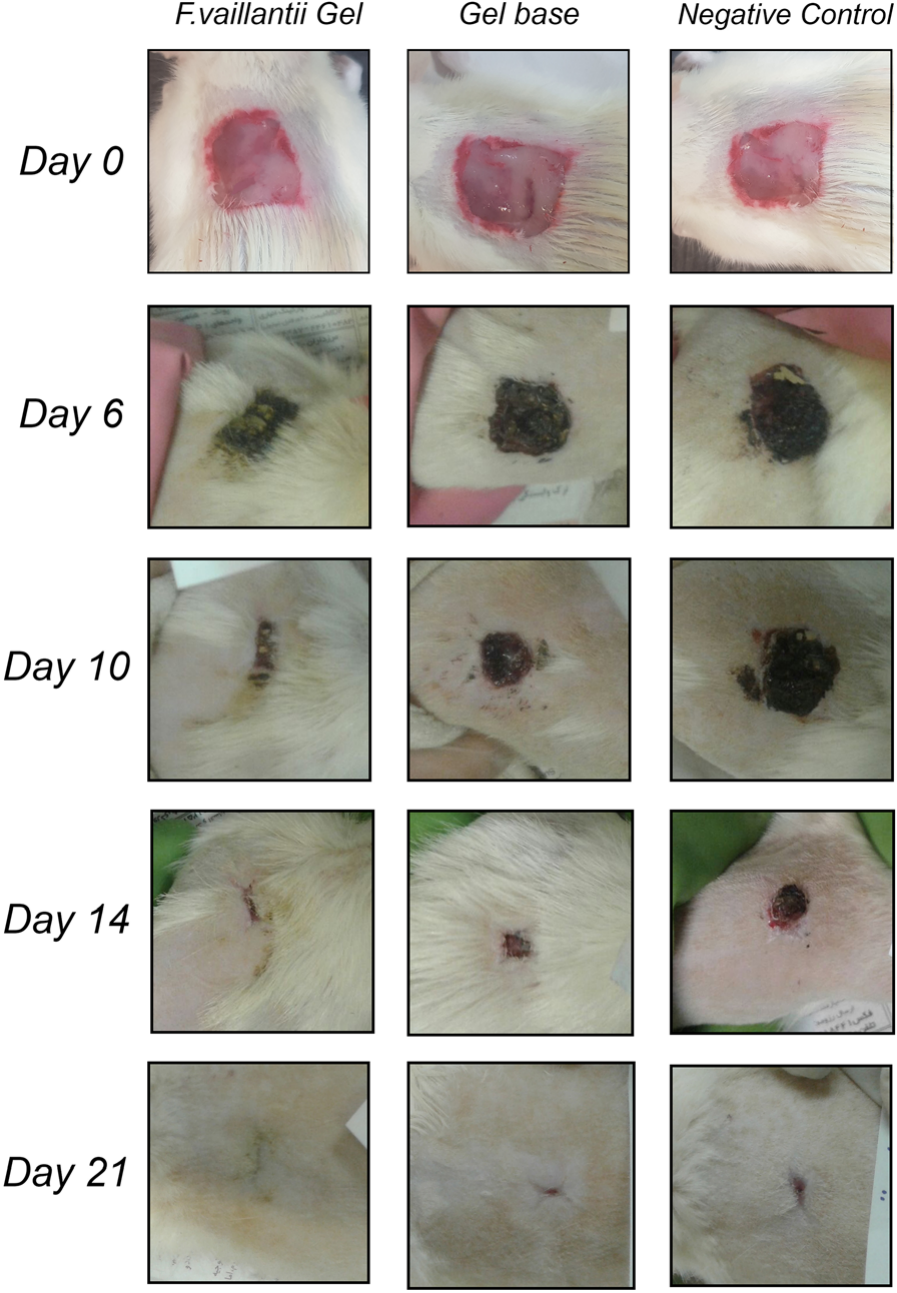
Photographs of the macroscopic observations of excision wound on days 6, 10, 14, 21. The rats were subjected to topical administration of *F.vaillantii* gel extract and gel base

**Fig. 3:**
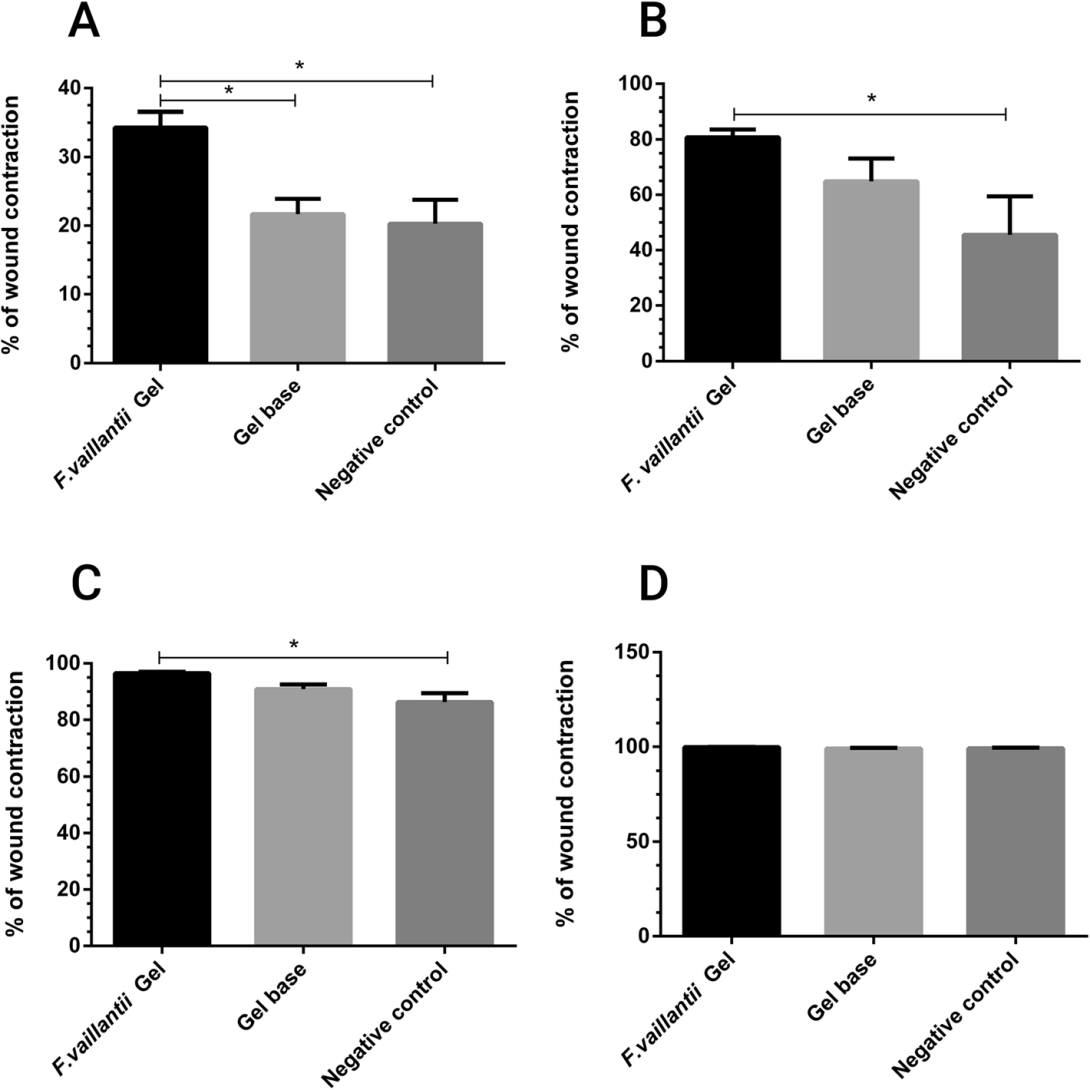
Percentage of wound contraction in the excision wound model upon administration of *F. vaillantii* gel formulation on different days; **a**) Day 6, **b**) Day 10, **c**) Day 14 and **d**) Day 21. Values are expressed as mean ± SEM of 5 animals in each group. *p < 0.05 versus negative control (one-way ANOVA, followed by Tukey’s test)

Wound contraction indicates the rate of increase of healed area. In other words, the faster the wound closure, the more effective would be the medication [26]. To study the rate of wound contraction and subsequent epithelialization, an excision wound model was established. The mean wound area was calculated in the control group as well as the treated groups as indices of wound closure within 21 days. Application of *F. vaillantii* gel formulation exhibited a considerable potential in wound healing activity. The macroscopic alterations of the wound area within 21 days are displayed in Fig. 2. On the day 6, *F.vaillantii* -treated group showed the highest percentage of wound contraction (35%) compared to both negative group (20%) and the group treated with gel base (20%) (Fig. 3). Furthermore, a significant difference (*p* < 0.5) was observed between *F. vaiilantii* gel-treated animals and the control on the days 10 and 14, and the wound was found to be almost healed on day 21 in the treated groups with no scar. Interestingly, the percentage closure of wound area was considerable on the 14 and 21 post-wounding days in animals treated with gel base. Noteworthy is that the wound closure rate was much slower in the treated rats on days 14 and 21 when compared with the control rats (Fig. 3).

To assess the breaking strength, the incision wound study was performed on the day 10 regenerated tissues. The results of the different parameters including maximum mechanical strength (f_max_, N); stress (N/mm^2^); absorbed energy (area up to f _max_; N × mm); deformation (mm); stiffness (N/mm) of the uniaxial tensile test are reported in Table 1. According to this table,topical application of either *F.vaillantii* formulated gel or gel base led to an increase in tensile strength compared to the negative control group after 10 days, although none of the tensile strength in the treated groups differed significantly from that of the control group. It is worthy to note that the groups receiving *F.vaillantii* hydrogel or gel base exerted more or less the same effect on the breaking strength of healing tissue. Indeed, very little development of breaking strength was observed following inclusion of the *F.vaillantii* extract. The mean of breaking strength in the healthy skin was much higher than that of the other groups.

**Table 1:** Stress (N/mm^2^), maximum mechanical strength (f_max_,N), absorbed energy (area up to f_max_(N *×* mm)), deformation (mm), stiffness(N/mm) 10 days post wound healing ^a^

Parameters	Stress (N/mm^2^)	f_max_ (N)	Area up to f_max_(N × mm)	Deformation (mm)	Stiffness(N/mm)
Groups					
*F.vaillantii* Gel	0.46 ± 0.06	19.08 ± 2.40	240.8 ± 30.63	22.49 ± 2.58	1.38 ± 0.15
Gel base	0.47 ± 0.05	19.99 ± 2.10	259.2 ± 53.09	23.34 ± 2.46	1.36 ± 0.10
Negative control	0.41 ± 0.08	18.03 ± 4.20	164.5 ± 68.26	14.52 ± 3.65	1.29 ± 0.14
Normal skin	0.77 ± 0.02^*^	34.52 ± 3.83^*^	600.3 ± 35.91^****^	30.49 ± 1.95^**^	1.49 ± 0.15

^a^Values are mean ± SEM of 5 animals in each group. *p < 0.05, **p < 0.01, ***p < 0.001, **** *p* < 0001 were considered significant compared to negative control group

### Histopathological study

**Fig. 4:**
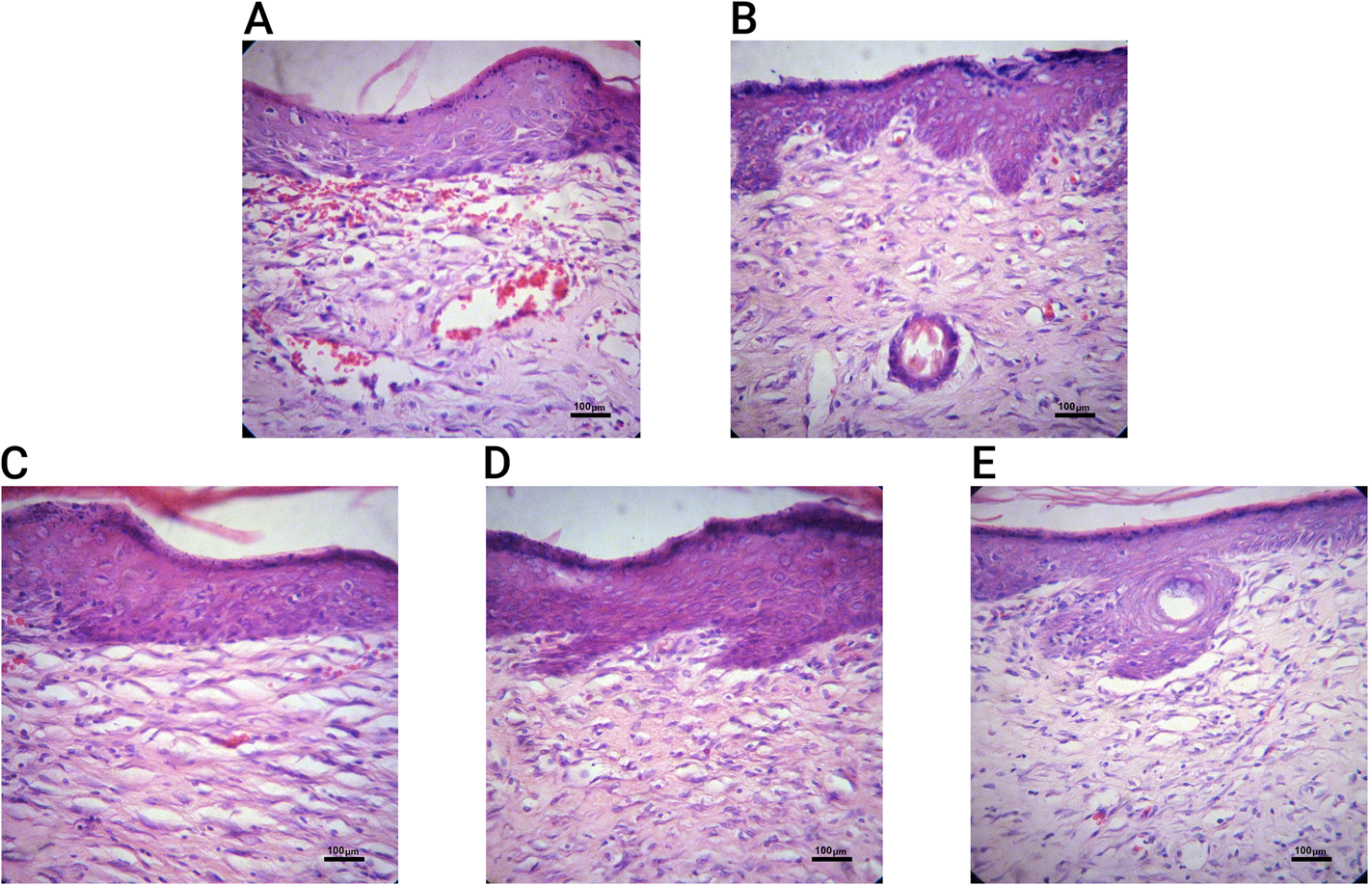
Microscopic photographs of histopathological evaluation of wound healing in the negative control, gel base and *F.vaillantii* gel extract administered. Skin sections display H& E stained in epidermis and dermis. The original magnification was 400×. **a**) Negative control: 21-old-wound tissue; **b**) Gel base group: 21-old-wound tissue treated with gel base; **c**),**d**),**e**) 21-old-wound tissue treated with *F.vaillantii* gel extract

**Fig. 5:**
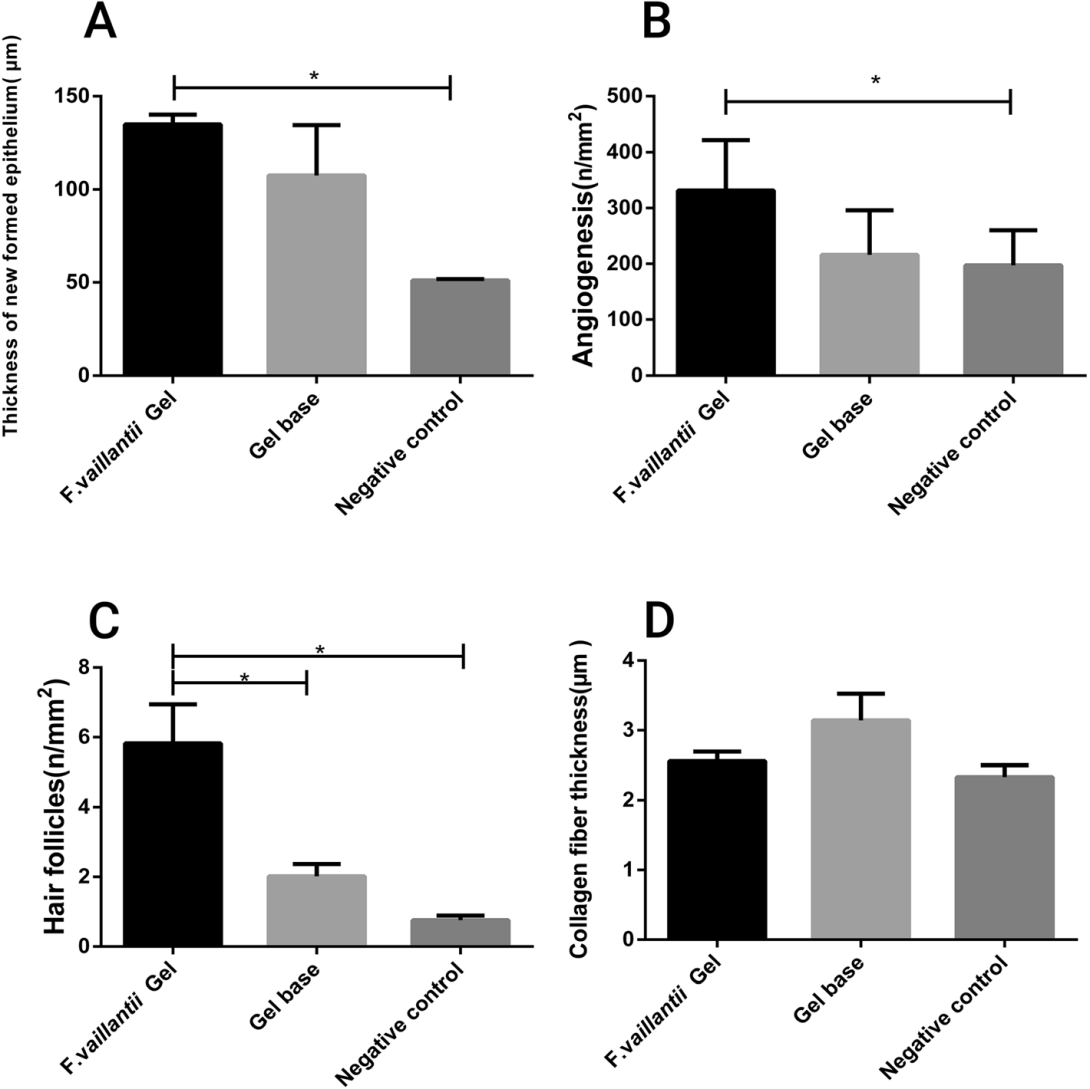
Comparison of **a**) epithelium thickness; **b**) angiogenesis (n/mm 2); **c**) number of hair follicles and **d**) collagen fiber thickness values of *F.vaillantii* gel extract, gel base and negative control on excision wound model. Values are mean ± SEM of 5 animals in each group. *p < 0.05 was considered significant

In our experiment on the untreated group (negative control), the thickness of the newly formed epithelium was significantly (*p* < 0.05) lower than that of the group submitted to the topical administration of *F.vaillantii* hydrogel (Fig. 4a and d). Following 21 days of surgery, collagen fibers, fibroblasts along with mature hair follicles were clearly established as shown in Fig. 4e. However, collagen fibrils were not well-organized and presented as sparse and irregular shapes in the negative control group (Fig. 4a). Inversely, in both gel base and *F.vaillantii* hydrogel groups, the dermis layer was regular with hair follicles and angiogenesis more than the control group (Figs. 4b, c, d and 5a, b, c). Nevertheless, the amount of collagen was almost the same in all experimental groups with a minute increase in the gel base treated group (Fig. 5d).

### Hydroxyproline content

**Fig. 6:**
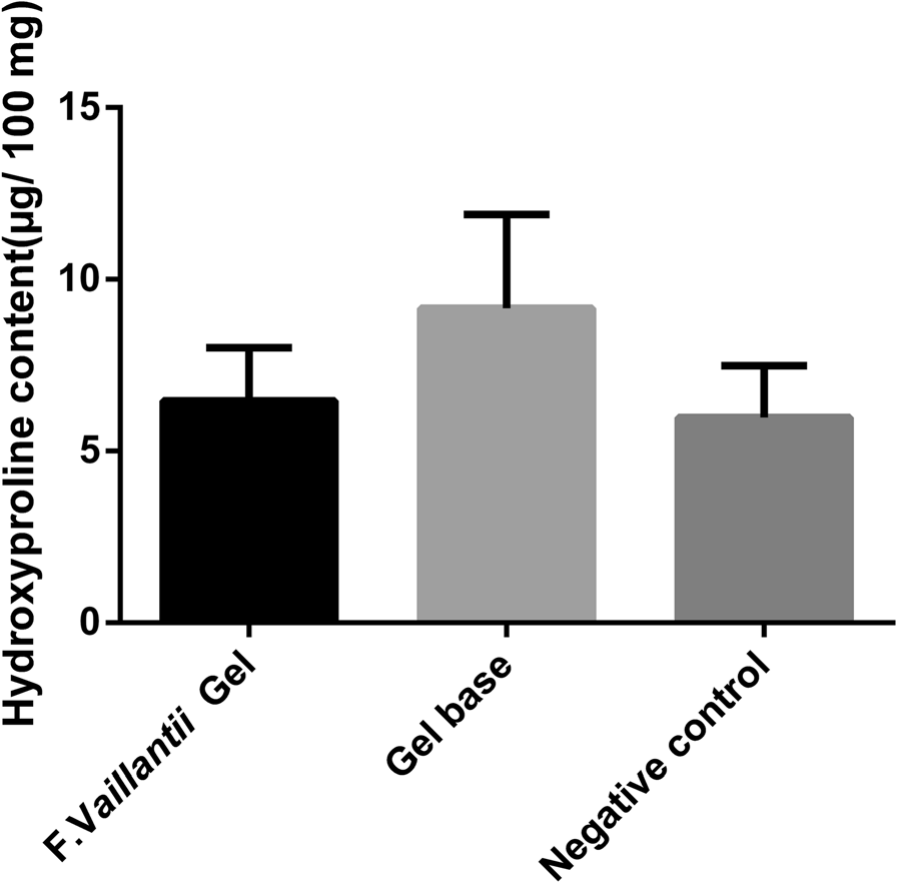
Hydroxyproline content (μg/100 mg skin) in different groups. Values represent mean ± SEM of 5 animals in each group

Collagen, a component of growing cells, is synthesized in the healing tissues. Thus, to further confirm the synthesized collagen in the wound areas, hydroxyproline content was determined (Fig. 6). Our results exhibited no significant difference in collagen deposition between *F.vaillantii* hydrogel-treated and the negative control groups, although the group receiving no treatment possessed the lowest content of hydroxyproline. Interestingly, gel base administration resulted in an insignificant increase in the amount of collagen.

### Quercetin content

HPLC chromatogram further acknowledged the presence of quercetin in the total extract of *F.vaillantii* (Additional file 1: Figure S1). The retention time (5.5 min) of the peak of quercetin was found in the total extract. The results demonstrated that the total extract of *F.vaillantii* contained 2.25% quercetin.

## Discussion

Breakdown in normal anatomic structure and function of the skin, termed skin wound, which could occur through several causes including physical injuries, may lead to the opening and disruption of the skin [27]. Wound healing is a dynamic process in which dermal and epidermal tissues regenerate as closely as possible to the normal status. A sequence of events occurs to repair the damage following injury. These events have been classified into several stages including inflammatory, proliferative and remodeling [28]. Cytokines are usually released in the inflammatory stage due to the phagocytosis of bacterial pathogens leading to the migration of the cells involved in the proliferative stage. A subsequent chain of events including angiogenesis, collagen deposition, granulation, tissue formation, epithelialization and wound contraction take place at the proliferative stage [29]. In the final stage, collagen remodeling takes place along the tension lines [30]. Non-toxic, novel and cost-benefit therapeutic agents that contribute to increased healing rate, hastened epithelialization, inhibition of bacterial infection and supporting tissue remodeling have gained a great deal of attention among researchers worldwide. Therefore, we herein attempted to make a hydrogel formulation containing 10% *F.vaillantii* total extract and applied it on an animal model of skin wound to verify the hypothesis that this formulation could demonstrate a distinguished treatment in healing wounds by providing an enhanced tissue repair.

Our results revealed that the topical administration of our hydrogel formulation using HPMC 4000 cP (2.5%) on a rat excision wound model leads to a significant acceleration in wound healing after 6, 10 and 14 days, a finding confirmed by an increased wound contraction compared to the negative control group. This enhanced potential of wound healing may be due to anti-inflammatory, antimicrobial and astringent properties of the plant, which are well documented in the literatures [31]. In this regard, our literature survey identified several phytochemicals including flavonoids, alkaloids, tannins and saponins present in the *F.vaillantii* total extract [11], which may be responsible for its wound contraction and enhanced rate of epithelialization 21 days post wound healing. This assumption is supported by our previous study indicating a considerable amount of flavonoids in the *F.vaillantii* total extract [32]. As flavonoids including quercetin are known to possess antioxidant and anti-inflammatory effects [33, 34], and according to the HPLC results performed here, confirming the presence of 2.25% of quercetin in total extract of *F.vaillantii*, the wound healing activity of *F.vaillantii* extract may be attributed to this property in the inflammatory phase. Furthermore, the alkaloids present in *F.vaillantii* are also responsible for its antimicrobial property [7, 35], which in turn leads to a better wound healing in the inflammatory phase.

Hydrogels exhibit several advantages as they provide the required moist environment to the wound area and also act as a suitable carrier for topical administration of substrates. Moreover, they cause a slow release of substances over time. What this information brings out noticeably is that hydrogel formulation can be a suitable candidate to promote wound healing. Thus, we prepared a hydrogel formulation with 10% total extract of *F.vaillantii* using 2.5% HPMC 4000 cP displaying an optimum consistency and spreadability. Consequently, the proper hydrogel spreading would assist in the uniform administration of the gel to the skin. Additionally, based on our results, our formulated herbal gel contributed to a faster wound healing compared to the negative control group. Surprisingly, collagen fiber thickness and hydroxylproline content appeared to be more or less similar but basically higher in the gel base than *F.vailla*ntii gel formulation -treated groups, which can necessarily be explained by the therapeutic effect of hydrogels alone. In addition, topical administration of *F.vaillantii* hydrogel was found to significantly increase the number of vessels as well as hair follicles in the gel-treated compared to the negative control wounds. Similar observations have also been reported by Xiao-bo Wu et al. (2012) who concluded that angiogenesis in granulation tissues results in improvement of circulation needed for supplying oxygen and nutrients vital for the healing process [36].

The establishment of an incision wound model needs to be further worked out in order to determine breaking strength, confirming the wound healing activity of 10% total extract hydrogel of *F.vaillantii*. In our study, control rats exhibited a wound breaking strength (area up to f_max_) of 164.5 ± 68.26 N/mm on the 10th post wound day, whereas gel base and the hydrogel extract-treated groups displayed no significant breaking strength (259.2 ± 53.09 and 240.8 ± 30.63 N /mm), respectively. These data are in agreement with the results of our excision model and highlight the role of the gel base in collagen production, which leads to stabilization of fiber formation and subsequent stable intra- and inter- molecular crosslinks [37, 38].

## Conclusion

The present study revealed that the hydrogel formulation containing *F.vaillantii* total extract (10%) promotes healing of epithelial wounds and enhances wound closure, number of hair follicles as well as angiogenesis at wound sites. This potency may be associated with the individual or synergistic effects of phytochemicals present in the total extract and provide evidence to some ethnomedicinal properties of *F.vaillantii*. The results of this study strongly encourage one to explore the efficiency of a hydrogel formulation containing a combination of several plant extracts in wound repair.

## Additional file

Additional file 1: **Figure S1.** Representative HPLC chromatogram of a) quercetin (5 μg/ml) b) total extract at UV detection λmax = 370 nm. (JPG 4060 kb)

## Data Availability

The datasets used and/or analyzed during the current study are available from the corresponding author on a reasonable request.

## Abbreviation

*F. vaillantii*: *Fumaria vaillantii*
*H&E*: *Hematoxylin and eosin*
*HPLC*: *High pressure liquid chromatography*
*HPMC*: *Hydroxypropyl methylcellulose*
