# Supplementary figures and images for: Topical hydrogel containing Fumaria vaillantii Loisel. extract enhances wound healing in rats
Source: BMC Complement Altern Med. 2019 Sep 12;19:254. doi: 10.1186/s12906-019-2645-y (PMC6739951; doi:10.1186/s12906-019-2645-y)

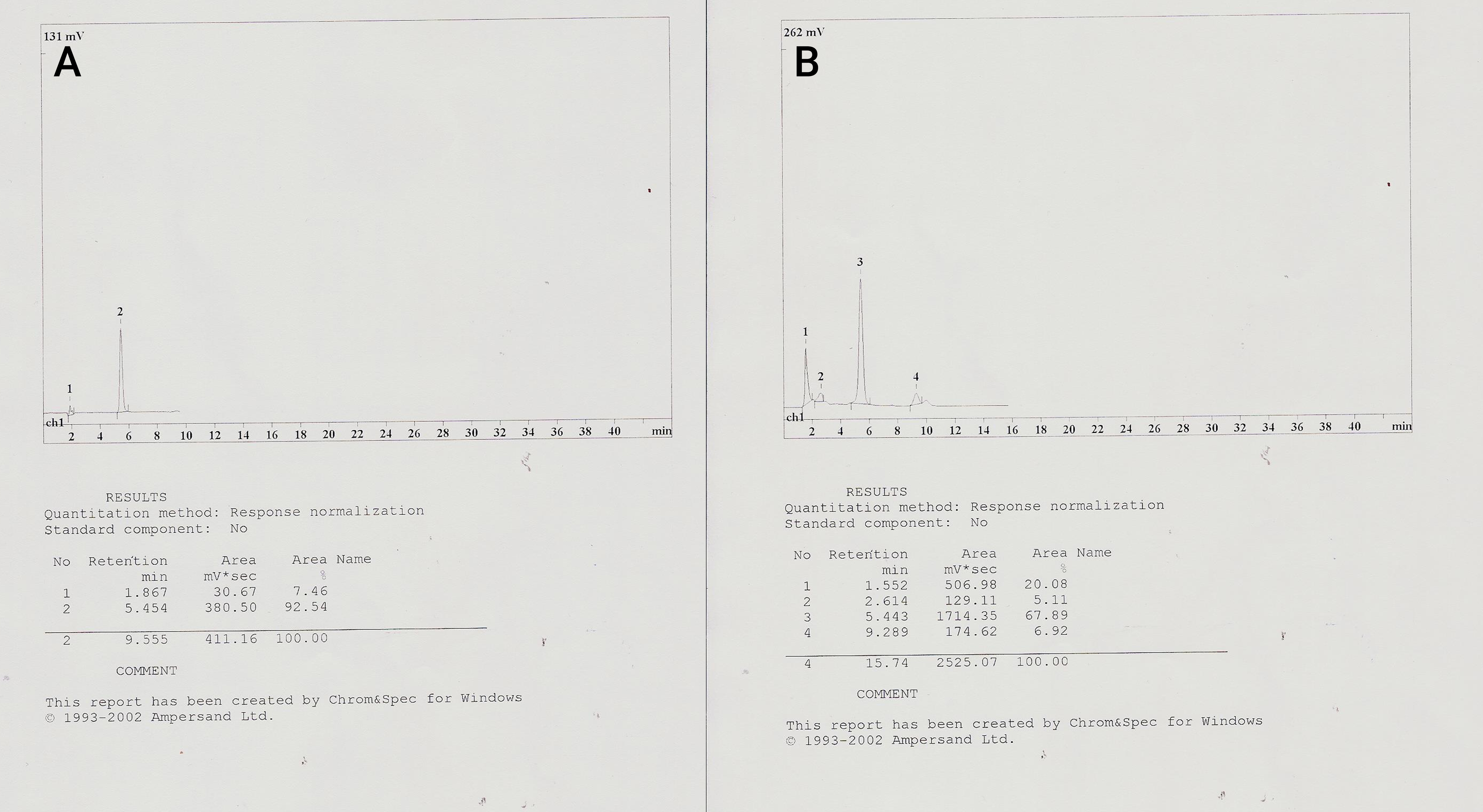

Supplement: Supplementary file 1 — Figure S1. Representative HPLC chromatogram of a) quercetin (5 μg/ml) b) total extract at UV detection λmax = 370 nm. (JPG 4060 kb) [file 12906_2019_2645_MOESM1_ESM.jpg]
